# JMCC plus receives first impact factor

**DOI:** 10.1016/j.jmccpl.2025.100480

**Published:** 2025-08-21

**Authors:** Davor Pavlovic, Rebekah Gundry

**Affiliations:** a Department of Cardiovascular Sciences, University of Birmingham, United Kingdom; b University of Nebraska Medical Center, Omaha, NE, United States of America

We are delighted to announce that *JMCC Plus* has received its inaugural Journal Impact Factor of 2.2, as released in the 2025 Journal Citation Reports. This important milestone is a testament to the quality of the research we publish and the commitment of our authors, reviewers, and editorial board.

Since our launch, *JMCC Plus* has aimed to provide an open-access, rigorous, and inclusive platform for the cardiovascular research community. The awarding of an impact factor marks a significant moment in our development—and coincides with a notable surge in manuscript submissions over the past 12 months.

As we continue to grow, we are eager to hear from our community. We welcome suggestions on how we can improve the ways we manage the journal, expand our outreach, and strengthen our editorial board. If you have ideas - or are interested in joining our editorial team - we would love to hear from you.

We are also pleased to share that *JMCC Plus* in addition to X, is now also active on the Bluesky social media platform (@jmccplus.bsky.social) and on LinkedIn (linkedin.com/company/jmcc-journals/). For this we thank our hard-working social media editors, Dr. Daniel Johnson, Dr. Yihua Bei and Ms. Olivia Baines, for their efforts in growing our digital presence, and we encourage readers to follow and engage with us online.

Finally, we invite researchers to propose new Special Issues that reflect emerging trends and challenges in cardiovascular science. Please feel free to contact the editorial team at d.pavlovic@bham.ac.uk or rebekah.gundry@unmc.edu with your ideas.

Thank you for your continued support—and for helping us reach this exciting milestone.

## CRediT authorship contribution statement

**Davor Pavlovic:** Conceptualization, Writing – original draft, Writing – review & editing. **Rebekah Gundry:** Writing – review & editing.

## Declaration of competing interest

The authors declare that they have no known competing financial interests or personal relationships that could have appeared to influence the work reported in this paper.

